# Curcumin Between Pleiotropic Potential and Translational Constraints

**DOI:** 10.3390/ijms27052212

**Published:** 2026-02-26

**Authors:** Alessandro Magini, Alessandro Datti

**Affiliations:** Department of Agricultural, Food, and Environmental Sciences, University of Perugia, 06121 Perugia, Italy

**Keywords:** curcumin, pleiotropy, nutraceuticals, pharmacokinetics, systems pharmacology, biomarkers, translational constraints

## Abstract

Curcumin is widely recognized for its anti-inflammatory and antioxidant properties; however, this conventional framing obscures a broader, complex, and mechanistically diverse pharmacology. Here, we advance a refined perspective that situates curcumin within a hierarchical and multilayered architecture shaped by the dynamic interplay of intrinsic chemical reactivity, metabolic transformation and exposure, and microbial modulation. From this standpoint, curcumin functions as a network-level modulator, producing context-dependent outcomes rather than uniform or linear responses. Consequently, its biological influence extends well beyond traditional paradigms, engaging pathways involved in xenobiotic metabolism, membrane transport, immune and metabolic signaling, and host-microbiome interactions, with downstream implications for drug disposition and biomarker interpretation. This complexity is further compounded by rapid clearance and limited systemic availability, although partially offset by the functional relevance of bioactive metabolites. Consistent with this pleiotropic model, clinical signals of curcumin activity tend to emerge in conditions characterized by multifactorial dysregulation, including metabolic, neurocognitive, and musculoskeletal disorders, as well as microbiome-associated alterations. Notably, human studies and meta-analyses frequently report divergent outcomes, with some trials demonstrating benefit and others showing substantial between-study heterogeneity. To reconcile these discrepancies, we advocate a *High Input*, *Rational Integration* paradigm that unifies experimental, preclinical, and clinical evidence obtained through logically rigorous and strictly consistent procedures applied across comprehensive, convergent, and reproducible datasets. Within the hierarchical organization of curcumin’s pharmacology, this approach enables the synthesis of mechanistic diversity within pharmacokinetic and physiological constraints and, more broadly, provides a coherent framework for interpreting pleiotropic bioactives in human studies.

## 1. Introduction

Curcumin, a polyphenolic metabolite of *Curcuma longa*, is historically anchored to anti-inflammatory and antioxidant properties [1,2,3]. This reputation, amplified across scientific and commercial sectors, crystallized conventional paradigms that overlooked broader biochemical reactivity, diverse cellular responses, and physiologically contingent effects. As a result, curcumin occupies a paradoxical position in biomedicine, being extensively studied and widely celebrated yet simultaneously constrained by conceptual oversimplifications [4,5,6]. This tension has long evoked both enthusiasm and skepticism, particularly when contradictory findings have emerged from mechanistic models that do not fit linear explanations or single-pathway frameworks [5,7,8].

Advances in chemical biology, systems pharmacology, and metabolic research now indicate that curcumin’s biological actions do not originate from a unified mechanistic basis. Rather, they stem from a multilayered interplay of electrophilic reactivity, biochemical perturbation, metabolic transformation, and interactions with xenobiotic-processing pathways [9,10,11]. Curcumin’s pleiotropy is therefore better understood as chemical and cellular events converging on shared regulatory nodes, such as NF-κB, Nrf2, AhR, and broader immune-modulating circuits [12,13,14,15], rather than as a simplistic *panacea-like* effect [5,8]. These nodes integrate inflammatory, metabolic, and environmental cues and are particularly responsive to pleiotropic molecules capable of simultaneously influencing multiple processes. Recognizing this architecture is essential to contextualize reported effects without the risk of overstating biological impact or therapeutic relevance.

Although the literature contains numerous examples documenting curcumin’s actions across biological systems, far fewer studies examine why these effects occur, which mechanisms remain chemically plausible under physiological conditions, or how these interactions control signaling outputs. Consequently, despite decades of investigation, curcumin remains a paradigmatic compound whose mechanistic diversity has yet to be fully integrated into a coherent framework linking molecular actions with biological outcomes [5,6,8,16,17,18].

This review seeks to address these gaps within a mechanistically grounded landscape. We examine the chemical, biochemical, and metabolic factors that collectively shape curcumin’s pharmacological profile and evaluate its capacity to modulate drug actions and xenobiotic processing. Through this integrated approach, our aim is not to advocate curcumin as a therapeutic agent but to provide a clearer foundation for experimental design, mechanistic interpretation, and fine-tuned nutraceutical applications.

## 2. Pleiotropic Architecture of Curcumin

**Figure 1:**
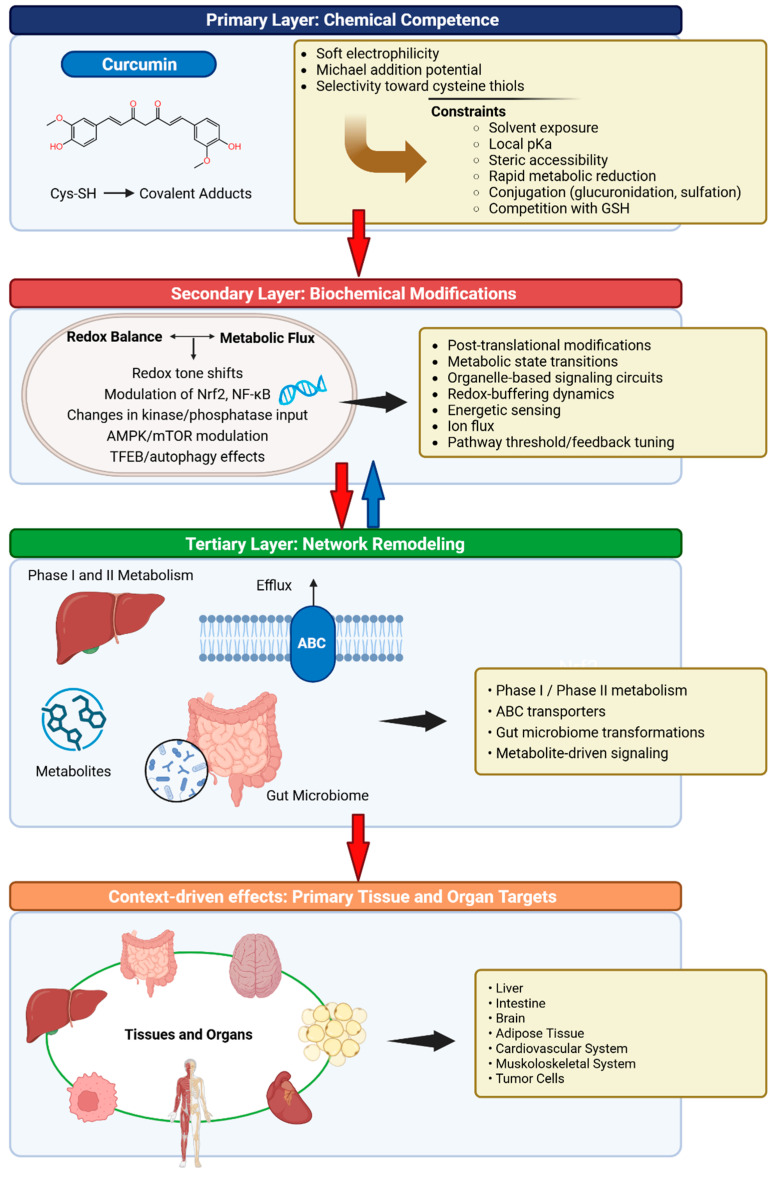
Pleiotropic architecture of curcumin. From the top, the diagram depicts the three hierarchical layers, namely, Chemical Competence, Biochemical Modifications, and Network Remodeling, together with their corresponding components, which collectively underpin the multifaceted effects of curcumin. Red arrows indicate the direction of the causal cascade described in the text, while the blue arrow denotes the feedback connection from layer 3 to layer 2. At the bottom, the primary tissue- and organ-specific targets are shown. Abbreviations: GSH, Glutathione; Nrf2, Nuclear factor erythroid 2-related factor 2; NF-κB, Nuclear factor kappa-light-chain-enhancer of activated B cells; AMPK, AMP-activated protein kinase; mTOR, Mechanistic target of rapamycin; TFEB, Transcription Factor EB; ABC, ATP-Binding Cassette (transporter). Created in BioRender. Magini, A. (2026) https://BioRender.com/71zoaah (accessed on 2 February 2026).

To disentangle sources of variability and enable integrative interpretation of the elements underlying curcumin’s biology, major determinants are organized into primary, secondary, and tertiary layers. In short, curcumin’s biological effects can be conceptualized as a hierarchical causal cascade with regulated feedback. At the first layer, intrinsic chemical competence, principally its modest electrophilic reactivity, defines the fundamental potential for molecular interaction. This potential is then filtered through a second layer of biochemical gating, including metabolism, conjugation, redox buffering, and transporter-mediated efflux, which collectively determine effective intracellular exposure. Only after passing these boundaries does curcumin engage the third layer, namely, network remodeling, where signaling architecture, inflammatory tone, and metabolic state determine whether perturbations translate into measurable biological outcomes. Importantly, this final layer can impart feedback on biochemical gating by, for example, altering enzyme expression or redox status, although the dominant direction of influence remains from chemistry to exposure to network response (Figure 1).

### 2.1. Primary Layer: Electrophilic Reactivity

Michael addition chemistry is a key driver of curcumin’s pleiotropic effects. This reactivity derives from two α,β-unsaturated carbonyl groups capable of engaging nucleophilic moieties, primarily thiols and, to a lesser extent, amines [19,20]. In this context, cysteine residues represent the dominant biological nucleophiles, and many regulatory proteins, including kinases, phosphatases, E3 ligases, and oxidoreductases, contain solvent-exposed or redox-sensitive thiols that can interact with small-molecule electrophiles. Thus, curcumin is best envisioned as a soft electrophile that preferentially reacts with soft nucleophiles like cysteine thiols [21].

The efficiency and site selectivity of these reactions are strongly shaped by the biochemical setting, as local pKa, steric accessibility, polarity, and solvent exposure collectively influence reaction likelihood and kinetics [8,19,21,22]. Under sub- to low-micromolar concentrations, commonly used in mechanistic studies, curcumin’s electrophilicity is relatively modest, such that only the most nucleophilic and solvent-accessible thiols are likely to undergo modification [8,21]. This limitation is further compounded in vivo by rapid metabolic reduction and conjugation, which markedly deplete the pool of circulating curcumin [5,23]. Consequently, its capacity to modify regulatory cysteines in proteins such as Keap1 or IKK is constrained [24,25], and interactions with other proteins harboring cysteine-responsive regulatory sites, for example, STAT3 and p300/CBP, remain more speculative [26,27].

Importantly, these electrophilic events do not function as deterministic *on/off* switches but are more plausibly interpreted as initiating or modulatory inputs within stress-sensing pathways. Like other soft electrophiles, curcumin alters the overall electrophilic and redox balance perceived by cellular stress sensors rather than selectively targeting a single enzyme. A representative example is the electrophilic perturbation of redox-sensitive cysteine residues in Keap1, which weakens rather than abolishes the Keap1-Nrf2 interaction. The extent and pattern of cysteine modification tune the probability of Nrf2 release, thereby enabling graded transcriptional activation of cytoprotective and antioxidant genes instead of a binary response [24,28].

In this manner, curcumin and related soft electrophiles sway stress-response signaling by shifting redox sensitivity in coordination with cellular thiol-buffering capacity and metabolic state, not by acting as high-affinity ligands for discrete regulatory sites. Consistent with this view, the symmetry and moderate electrophilicity of curcumin favor interactions with multiple nucleophilic sites at low specificity, making a single-target, high-affinity mechanism unlikely [5,29,30,31].

Therefore, within the biochemical environment typical of most cells, curcumin’s electrophilic reactivity functions primarily as an upstream modulator of stress-response pathways rather than a direct inhibitor of specific molecular targets. Curcumin is present only transiently and at very low levels due to rapid reduction and extensive conjugation. Moreover, this constraint operates in parallel with substantial thiol-buffering capacity (e.g., glutathione [GSH]), which competes for electrophilic species. Together, these factors markedly restrict curcumin’s ability to form covalent adducts with signaling proteins. As a result, its electrophilic activity supports only a narrow range of biologically meaningful interactions, reinforcing the context-dependent nature of associated biological effects [5,8].

### 2.2. Secondary Layer: Biochemical Modifications

Beyond direct chemical reactivity, curcumin influences cellular signaling through broader biochemical perturbations that reshape redox balance, post-translational regulation, and metabolic fluxes. Unlike Michael addition, these effects do not arise from engagement with a single molecular target but from curcumin’s capacity to shift the operating state of regulatory networks [11,25]. As a result, curcumin affects multiple signaling cascades, including inflammatory, stress-response, and metabolic pathways, largely through indirect mechanisms that unfold downstream of its initial electrophilic interactions [14,32,33].

Central to these perturbations is the influence on redox-sensitive signaling hubs. For example, curcumin can influence Keap1-Nrf2 and NF-κB pathways not only through covalent modification of reactive cysteine residues but also by altering the local redox environment [24,33,34]. Modest changes in oxidative or electrophilic tone can bias the activity of these sensors, promoting adaptive responses such as antioxidant gene induction or attenuation of pro-inflammatory signaling, consistent with curcumin’s pleiotropic and context-dependent biological actions.

In parallel, curcumin influences enzymes that govern post-translational regulatory processes, including kinases, phosphatases, and acetyltransferases, thereby evoking biochemical shifts that can amplify or dampen cellular responses depending on network state and stimulus intensity [34,35,36]. The magnitude and directionality of these effects depend on curcumin concentration, cellular redox status, and competition from endogenous nucleophiles, underscoring the importance of biochemical and biological context when interpreting mechanistic observations [37,38,39,40].

Curcumin also shapes metabolic signaling by modulating key nodes involved in energy homeostasis and nutrient sensing. Specifically, interactions with AMPK- and mTOR-associated pathways influence cellular energy balance, autophagy, and stress-adaptation responses [41,42,43]. For example, these regulatory inputs converge on the lysosomal-autophagy axis, where TFEB functions as a central transcriptional controller of adaptive programs. Consistent with this, curcumin has been reported to promote TFEB nuclear translocation and restore disease-associated cellular phenotypes [44]. While such effects are commonly observed in vitro at low- to mid-micromolar concentrations, their translation in vivo depends on systemic exposure, tissue distribution, and the local biochemical context [45]. Overall, these biochemical perturbations recapitulate the role of curcumin as a systems-level modulator rather than a classical inhibitor or activator. Within this framework, upstream electrophilic interactions initiate network-level adjustments that propagate through signaling circuits, with downstream biochemical outcomes arising from curcumin’s initial reactivity and subsequently conditioned by metabolic transformation and cellular network complexity.

### 2.3. Tertiary Layer: Network Remodeling and Regulatory Dynamics

The biochemical gating established at the secondary layer is further shaped by Phase I and Phase II metabolic events, which critically determine curcumin’s chemical competence, bioavailability, and downstream signaling potential [46,47,48]. Phase I and Phase II metabolic pathways provide the principal biochemical framework through which curcumin is processed in vivo. Phase I reactions, largely mediated by the cytochrome P450 superfamily of reductive and oxidative enzymes, introduce or modify functional groups on the parent molecule, while Phase II conjugation pathways, driven by enzymes such as UDP-glucuronosyltransferases and sulfotransferases, couple these intermediates to glucuronic acid or sulfate, markedly increasing hydrophilicity and facilitating rapid clearance. In vivo, curcumin undergoes rapid reduction to form tetrahydro- and hexahydro-derivatives, along with extensive glucuronidation and sulfation [49]. As these reactions generally proceed at high rates, only a limited fraction of the parent compound remains unconjugated and chemically active. With fewer molecules available to modify nucleophilic residues in regulatory proteins, metabolic processing becomes a primary determinant of which molecular interactions are realistically accessible [8,50,51,52].

These metabolic events operate alongside membrane transport mechanisms that further impact curcumin’s cellular exposure. ABC transporters, particularly P-glycoprotein (P-gp), interact with curcumin and contribute to its complex pharmacokinetic behavior. Notably, rather than serving primarily as an efflux barrier, curcumin has been repeatedly shown to inhibit P-gp activity and, in some models, downregulate its expression, thereby enhancing intracellular retention of P-gp substrates and modulating multidrug resistance phenotypes [53,54]. Nevertheless, transporter interactions occur in parallel with rapid metabolic reduction and conjugation, together defining a narrow temporal and spatial window in which unconjugated curcumin can engage electrophile-sensitive targets. Thus, cellular exposure is not merely governed by external dosage but also by the integrated interplay between metabolism and transport, which collectively constrains the scope and persistence of electrophilic interactions [8,46,53].

At a higher organizational level, interactions with the gut microbiome introduce an additional layer of metabolic plasticity through a spectrum of biochemical transformations. Microbial enzymes catalyze reductive, deconjugative, and other biotransformations of curcumin, generating metabolites with distinct bioactivities and pharmacological relevance [55,56,57,58]. In parallel, curcumin modulates microbial composition and functional capacity, establishing a bidirectional axis in which microbial ecology and curcumin metabolism jointly shape systemic physiology [55,56,59]. This interplay has emerged as a focal point of investigation, driven by evidence that curcumin-derived metabolites and degradation products play a substantial role in shaping overall pharmacological behavior [60]. Accumulating data further implicate these metabolites in diverse signaling pathways and regulatory networks associated, for example, with immune regulation [61], metabolic homeostasis [62], neuroprotection and neuroinflammatory signaling [32], and epigenetic remodeling [63].

Microbial metabolism and host-microbiome cooperation therefore disrupt any linear relationship between curcumin exposure and pharmacological outcome across tissues. In this context, curcumin-derived metabolites may partially compensate for the rapid clearance and low systemic availability of the parent compound by engaging distinct receptors, transcriptional regulators, or epigenetic modifiers, thereby enabling pathway-specific modulation in settings where free curcumin is scarce [55,64,65]. Moreover, interindividual variability in gut microbiota composition likely contributes to substantial heterogeneity in both the nature and magnitude of these effects, complicating both experimental reproducibility and translational development.

This metabolic dynamic provides a mechanistic basis for the long-recognized paradox whereby curcumin, despite poor bioavailability, is nevertheless associated with a broad spectrum of biological effects in vivo [55,56]. Importantly, it also highlights opportunities for the identification of analogs or metabolite-inspired scaffolds more effectively positioned for specific pharmacological applications [66,67].

### 2.4. Context-Dependent Pleiotropy

A descriptive outline of the three hierarchical determinants discussed earlier provides only a partial understanding of curcumin’s pleiotropic behavior. These determinants, namely, chemical competence (i.e., availability of unconjugated, electrophile-active curcumin), biochemical gating (i.e., metabolic transformation, redox buffering, transporter activity), and network responsiveness (i.e., capacity of signaling circuits to amplify or constrain electrophilic cues), are not fixed properties. Rather, they shift with physiological state, developmental stage, inflammatory tone, nutritional status, and disease-related remodeling.

As these variables fluctuate, their interplay continually reshapes which molecular targets are accessible, how efficiently electrophilic reactions occur, and which downstream pathways are activated. Thus, the heterogeneous effects of curcumin do not stem from an inherent multitarget nature but from how biological backdrops assemble and recalibrate these determinants. Even subtle differences in metabolic flexibility, redox buffering, or signaling architecture can reorganize the response landscape, producing markedly different outcomes across cell types, tissues, and pathological states. This framework, supported by extensive experimental evidence, accounts for the breadth of curcumin’s actions, extending far beyond conventional anti-inflammatory and antioxidant paradigms [5,8,11,68].

Macrophages and glutathione (GSH)-based redox buffering provide a clear example of context dependence. In LPS-activated RAW264.7 cells, blocking GSH synthesis with buthionine sulfoximine (BSO) markedly increases curcumin’s potency toward NF-κB suppression, indicating that under normal conditions, GSH scavenges reactive curcumin metabolites and limits their interaction with electrophile-sensitive targets. Conversely, elevation of intracellular GSH with N-acetylcysteine decreases curcumin’s efficacy, demonstrating that robust GSH-dependent antioxidant buffering can restrict curcumin’s oxidative activation and the resulting downstream signaling outcomes [69]. Upon inflammatory activation, however, elevated ROS, increased thiol exposure within Keap1 or IKK complexes, and a more permissive signaling architecture collectively amplify curcumin’s effects, suppressing NF-κB–driven outputs and inducing cytoprotective programs [24,70,71].

Transformation status offers another well-supported case. Cancer cells, characterized by oxidative stress, altered membrane transport, and metabolic rewiring, show reduced conjugation capacity and increased accessibility of electrophile-sensitive targets [72,73,74,75]. Under these conditions, levels of unconjugated curcumin within the 20 mM range readily engage regulators that tilt signaling toward cell cycle arrest, autophagy, or apoptosis [76,77,78], whereas non-transformed (normal) cells, with stronger redox and metabolic gating, are generally less sensitive to curcumin treatment [79].

Metabolic tissues further illustrate the context-dependent nature of curcumin’s pleiotropic signaling. Curcumin engages pathways governing energy homeostasis and nutrient sensing, including AMPK- and mTOR-associated networks, but the magnitude and downstream consequences of these interactions vary with physiological state rather than reflecting a uniform response. While curcumin can activate AMPK under basal conditions, multiple studies indicate that its metabolic effects are amplified or qualitatively redirected in settings characterized by energetic imbalance or metabolic stress, such as high-fat feeding, insulin resistance, or lipid overload [45,80,81,82,83].

For example, in diet-induced hepatic steatosis, curcumin enhances AMPK activity and suppresses lipogenic programs more robustly than in a metabolically healthy liver, contributing to improved lipid handling and stress adaptation [84]. Similarly, in skeletal muscle, curcumin modulates glucose uptake and lipid metabolism through AMPK-ACC signaling, with stronger functional consequences observed under conditions of nutritional excess or metabolic challenge [85]. Moreover, under intense physical activity, curcumin influences the PI3K/Akt-AMPK axis to support metabolic adaptation marked by enhanced glycogen synthesis, a lower AMP/ATP ratio, and reduced lactate accumulation, thereby attenuating muscle fatigue in vivo [86].

An additional layer of regulatory evidence emerges in the nervous system, where specialized redox microdomains, dynamic glutathione pools, and activity-dependent signaling circuits collectively govern responses to redox-active perturbations [87,88]. Under non-stressed (basal) conditions, robust antioxidant defenses and tightly regulated redox buffering generally limit curcumin’s interaction with electrophile-sensitive targets. Nevertheless, curcumin can elicit measurable biochemical effects even at baseline, activating stress-responsive pathways and modulating antioxidant systems in the absence of overt cellular stress. This behavior is consistent with hormetic regulation of redox networks rather than a role strictly confined to pathological states [89,90]. In contrast, during metabolic stress, neuroinflammatory states, or excitotoxic challenge (conditions that elevate oxidative burden and transiently expose redox-sensitive sites), curcumin’s effects on neuronal resilience, stress signaling, and adaptive responses are markedly enhanced. In experimental models of neurotoxicity and ischemic injury, curcumin attenuates reactive oxygen species and endoplasmic reticulum stress, activates AMPK-linked protective pathways, and suppresses inflammasome activation, thereby limiting neuronal damage and promoting survival [91,92]. Moreover, evidence from neurodegenerative disease models, including Alzheimer’s disease, Parkinson’s disease, and ischemic stroke, indicates that curcumin modulates multiple signaling axes (e.g., NF-κB, BDNF/TrkB, GSK-3β, MAPK) involved in neuroprotection. These actions are accompanied by preservation of synaptic integrity and neuroplasticity, improved mitochondrial function, enhanced amyloid-β clearance, inhibition of tau hyperphosphorylation, metal ion chelation, and overall neuronal survival under challenged conditions [14,92,93,94,95]. Thus, taken together, these findings underscore that curcumin’s neurophysiological effects are not uniform but arise selectively when neural networks are challenged, recapitulating a context-dependent modulation of stress-responsive circuits.

In conclusion, curcumin does not impose a fixed biological program. Rather, the biological consequences of treatment are filtered through, and reshaped by, the hierarchical architecture and heterogeneity of each cellular environment. Hypoxic niches, lipid-rich microdomains, and antioxidant-poor regions of inflamed tissue create local conditions that enhance curcumin’s effective activity by promoting membrane partitioning, reducing conjugation, or transiently exposing reactive targets. Conversely, highly reducing or efflux-enriched compartments sharply curtail engagement, generating intratissue variability despite uniform systemic exposure. Altogether, these examples, supported by a broad experimental literature, highlight a pharmacological potential that extends well beyond traditional classifications.

## 3. Curcumin as a Modulator of Drug Actions

**Figure 2:**
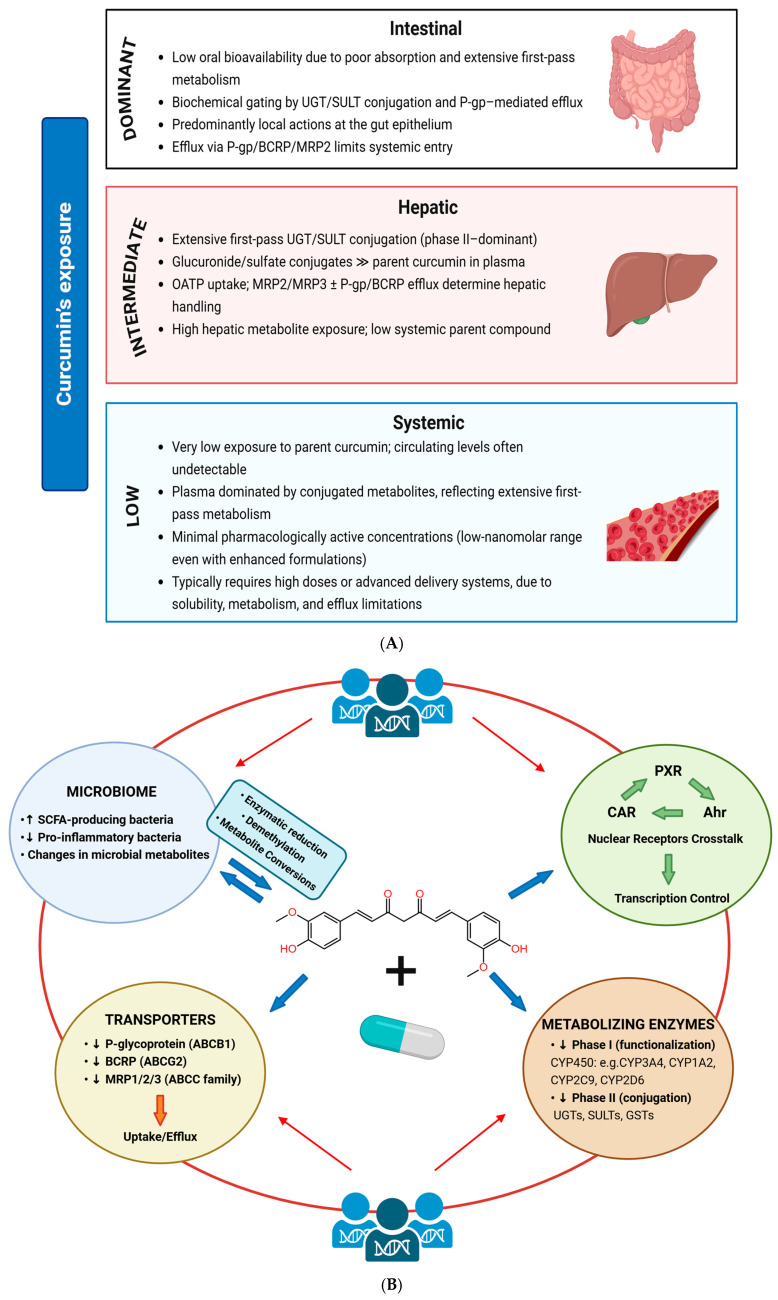
Determinants of curcumin disposition and action. (**A**) Local and systemic exposure of curcumin. Intestinal: Curcumin exhibits low oral bioavailability due to poor absorption and extensive first-pass metabolism. Biochemical gating by UGT/SULT conjugation and P-gp–mediated efflux limits uptake, leading to predominantly local actions at the gut epithelium. Efflux via P-gp/BCRP/MRP2 further reduces systemic entry. Hepatic: First-pass UGT/SULT conjugation (phase II-dominant) predominates, resulting in glucuronide/sulfate conjugates far exceeding parent curcumin in plasma. Hepatic disposition reflects OATP-mediated uptake and MRP2/MRP3 with ± P-gp/BCRP efflux, resulting in high hepatic metabolite exposure and low systemic parent compound. Systemic: Parent curcumin exposure is very low, often undetectable after oral dosing; plasma is dominated by conjugated metabolites (glucuronides and sulfates), reflecting extensive first-pass metabolism. Systemically, pharmacologically active parent concentrations are minimal, typically low-nanomolar, even with enhanced formulations, so meaningful systemic exposure generally requires high doses or advanced delivery systems. Created in BioRender. Magini, A. (2026) https://BioRender.com/3iyrjjr (accessed on 2 February 2026) (**B**) Mechanistic drivers of curcumin-mediated drug modulation. Schematic overview of bidirectional interactions among curcumin and the gut microbiome, drug transporters, xenobiotic metabolizing enzymes, and nuclear receptor signaling networks; human genetic heterogeneity (icons at top and bottom) contributes additional variability to these processes. Abbreviations: SCFAs, Short-chain fatty acids; BCRP, Breast Cancer Resistance Protein; MRP, Multidrug Resistance-Associated Protein; P-gp, P-glycoprotein; OATP, Organic Anion Transporting Polypeptide; PXR, Pregnane X Receptor; CAR, Constitutive Androstane Receptor; AhR, Aryl Hydrocarbon Receptor; CYP, Cytochrome P450 enzymes; UGT, UDP-Glucuronosyltransferase; SULT, Sulfotransferase; GST, Glutathione S-Transferase. Created in BioRender. Magini, A. (2026) https://BioRender.com/jwb61hu (accessed on 2 February 2026).

A reflection of the contingent and versatile behavior of curcumin is its capacity to modulate drug actions through an integrated network of mechanisms that include enzymatic inhibition or induction, transporter modulation, nuclear receptor crosstalk, and microbiome-mediated metabolism. These effects are inherently context-dependent, emerging from concentration, formulation, metabolic capacity, and tissue-specific exposure. Although not a conventional therapeutic agent, curcumin exhibits integrated pharmacokinetic and pharmacodynamic modulation in that it can influence drug metabolism and transport and alter the bioavailability of co-administered compounds, including its own absorption profile, while also affecting cell-signaling pathways, inflammatory responses, and redox balance. Thus, curcumin represents a paradigmatic model for understanding how chemically and biologically multifaceted nutraceuticals can modify drug disposition, safety, and efficacy (Figure 2A,B).

### 3.1. Modulation of Drug-Metabolizing Enzymes

Curcumin can directly interact with drug-metabolizing enzymes involved in both Phase I and Phase II biotransformation. Among Phase I enzymes, cytochrome P450 (CYP) isoforms are inhibited primarily through reversible active-site binding and, in some cases, time-dependent mechanisms, either competitively (CYP3A4 and CYP1A2) or non-competitively (CYP2C9 and CYP2D6), with the greatest inhibitory potency reported for CYP3A4, CYP2C9, and CYP2C19. Notably, these inhibitory effects occur predominantly within the low micromolar range—above typical systemic concentrations—suggesting that pharmacologically relevant interactions, especially with orally administered CYP3A4 substrates, most likely arise in the intestinal mucosa during absorption, where local exposure is high, rather than through hepatic metabolism [96,97,98].

Curcumin similarly affects Phase II enzymes, including UDP-glucuronosyltransferases (UGTs) and sulfotransferases (SULTs). As observed in human cell systems and enzyme preparations, curcuminoids inhibit both UGT- and SULT-mediated conjugation in vitro with significant and moderate impact on sulfation and glucuronidation, respectively, underscoring potential to alter clearance of co-administered drugs and xenobiotics [97,99].

These inhibitory interactions are strongly dose- and exposure-dependent. Although in vitro studies typically apply concentrations exceeding physiological levels due to limited systemic bioavailability, luminal concentrations in the intestine after oral administration can be substantially higher than circulating levels, making local enzyme inhibition feasible [97]. In addition, inhibitory thresholds reported for SULT1A1 in the liver and extrahepatic tissues fall within or below peak plasma concentrations following very high-dose supplementation (≈4 g), suggesting that systemic effects are likely achieved under aggressive dosing conditions [100].

Compounding this complexity, curcumin has also been shown to induce Phase II enzyme expression—such as UGT1A1 and UGT1A6—by transcriptional regulation [99], revealing a dual inhibitory and inductive influence associated with a context-dependent engagement with drug metabolism.

### 3.2. Crosstalk with Nuclear Receptors

The involvement of curcumin with nuclear receptors builds an additional mechanistic layer to its biological and pharmacological profile. Curcumin has been shown to modulate the pregnane X receptor (PXR) [101,102], the aryl hydrocarbon receptor (AhR) [103,104], and, potentially, the constitutive androstane receptor (CAR) [105], which are central regulators of xenobiotic sensing and detoxification through transcriptional activation of genes encoding phase I and II drug-metabolizing enzymes, as well as uptake and efflux transporters [106]. These interactions may produce non-linear outcomes due to ligand promiscuity, extensive receptor crosstalk with downstream signaling pathways, and tissue-specific expression patterns [107,108], thereby extending curcumin’s influence on xenobiotic disposition well beyond direct enzymatic modulation. Moreover, curcumin’s activity at these receptors is highly context-dependent, since factors such as inflammatory signaling, cellular redox state, and competing ligands can shift its role from partial agonist to functional antagonist, likely through changes in receptor conformation and coactivator recruitment [103,104].

The implications of curcumin’s interaction with nuclear receptors are supported by indirect evidence from animal models. Although these studies do not directly identify PXR, CAR, or AhR as the upstream mediators, the observed alterations in CYP and transporter expression remain consistent with nuclear receptor-dependent regulation. For example, in mice with non-alcoholic fatty liver disease (NAFLD) induced by a high-fat/high-fructose diet, curcumin restored hepatic expression of CYP3A and CYP7A via the Nrf2-FXR-LXRα axis and potential crosstalk with additional nuclear receptor pathways [109]. Likewise, repeated oral curcumin administration in rats modulated intestinal, hepatic, and renal CYP3A and P-glycoprotein expression, producing measurable changes in the pharmacokinetics of peroral celiprolol and midazolam in a manner compatible with receptor-regulated xenobiotic disposition in vivo [110]. In another study, curcumin reduced everolimus bioavailability in rats by enhancing CYP3A-mediated metabolism despite concurrent P-glycoprotein inhibition [111].

Taken together, these observations indicate that nuclear receptor modulation by curcumin is biologically plausible in vivo and may contribute to exposure-dependent variability in drug pharmacokinetics.

### 3.3. Impact on Drug Transporters

Modulation of drug transporters plays a critical role in the absorption, distribution, and elimination of xenobiotics, representing a mechanistic layer distinct from direct enzyme inhibition or nuclear receptor-driven transcription. Beyond its effects on drug-metabolizing enzymes, curcumin interacts with major efflux and uptake systems, most prominently P-glycoprotein (ABCB1/P-gp) and breast cancer resistance protein (ABCG2/BCRP), and, to a lesser extent, organic anion-transporting polypeptides (OATPs) and related solute carriers [112]. Curcumin inhibits ABCB1 in various models, including primary rat hepatocytes [113], Caco-2 intestinal monolayers [114], and drug-resistant human carcinoma KB-V1 cells [115], leading to increased intracellular accumulation of transporter substrates and chemosensitization. Curcumin further inhibits ABCG2/BCRP, with effects demonstrated at the blood-brain barrier in ex vivo rat brain capillaries [116] and enhanced oral bioavailability of the BCRP substrate sulfasalazine in both mice [116] and a human crossover study [117], confirming clinical BCRP inhibition and revealing non-linear pharmacokinetics.

However, translational relevance remains context-dependent and challenging to assess due to four interconnected factors: (i) although P-gp inhibition is widely reported, most in vitro studies employ low-micromolar curcumin concentrations that exceed those achievable systemically after oral intake [118]; (ii) rapid metabolic clearance constrains systemic transporter modulation, making interactions in high-exposure compartments, particularly the intestinal lumen, more plausible than at distal sites such as the blood-brain barrier [32,119]; (iii) inhibitory effects on BCRP and MRP2 (ABCC2) depend on dose, substrate specificity, timing, co-administered compounds, and interindividual variability, which can alter the fate of conjugated metabolites and favor their systemic persistence over excretion [120]; and (iv) formulations of curcumin, as nano-encapsulated and lipid-based solutions, yield higher luminal or hepatic concentrations than conventional powders, increasing the potential of physiologically significant transporter engagement [121,122]. Taken together, curcumin-mediated modulation of drug transport is conditional, hierarchical, non-uniform, and most pronounced in anatomical sites with the highest exposure, such as the gastrointestinal tract.

### 3.4. Microbiome-Mediated Modulation

While curcumin-microbiome bidirectionality has been described earlier, its direct implications for drug pharmacology warrant specific attention. Curcumin-induced shifts in microbial composition can alter luminal metabolism of co-administered drugs, influence hepatic enzyme expression through microbiota-derived signaling molecules, and modulate transporter regulation via bile acid-dependent nuclear receptor pathways [55,56,123]. In parallel, microbial β-glucuronidases can deconjugate curcumin glucuronides, sustaining a local pool of active curcumin and extending the window for intestinal interaction with drugs absorbed in the same compartment [56,57].

Importantly, these effects are highly variable, given that inter-individual differences in microbiome structure can lead to unpredictable changes in drug exposure and therapeutic response [55,123,124]. Microbiome-mediated modulation therefore represents an additional and under-recognized source of uncertainty in curcumin-drug interactions, one that likely contributes to heterogeneity across preclinical and clinical findings.

### 3.5. Mechanistic and Clinical Complexity of Curcumin-Drug Interactions

Curcumin influences multiple pathways involved in drug disposition, including CYP3A, P-glycoprotein (P-gp), and breast cancer resistance protein (BCRP). As shown in Table 1 (and references therein), preclinical studies often report consistent interaction patterns, for example, increased systemic exposure following BCRP inhibition (e.g., sulfasalazine) or enhanced absorption associated with P-gp inhibition (e.g., celiprolol). However, despite these relatively well-characterized mechanisms in experimental models, human studies are frequently lacking, and translation to clinical care remains uncertain for many drugs.

Discrepancies across studies commonly arise from differences in curcumin dose and formulation, microbiome composition, substrate specificity (e.g., CYP3A versus P-gp dependence), and the relative contribution of metabolic versus transporter pathways, in addition to disease- and context-specific clinical factors. These sources of variability, particularly the balance between local (intestinal) and systemic exposure, underscore the need for carefully designed clinical evaluations before firm recommendations can be established, especially for orally administered and narrow-therapeutic-index medications. For CYP3A substrates given orally, opposite effects have been observed depending on whether intestinal inhibition or hepatic induction predominates [110], highlighting the hierarchical nature of curcumin’s pleiotropy.

Overall, while certain curcumin-drug combinations suggest potential therapeutic opportunities, most interactions remain unpredictable without controlled monitoring. In some cases, such as tacrolimus, this uncertainty necessitates careful reconciliation of prescribed drug therapy with the use of turmeric or curcumin as a supplement or dietary component for unrelated indications (e.g., anti-inflammatory purposes).

## 4. Translational Landscape of Curcumin Beyond Redox and Inflammation

### 4.1. Background

The pleiotropic pharmacology and chemical versatility of curcumin have prompted sustained efforts to translate mechanistic insights into clinical applications [135]. To date, 393 human studies registered on ClinicalTrials.gov (accessed on 5 January 2026) span metabolic, oncological, neurocognitive, gastrointestinal, and supportive-care contexts. Yet, despite this apparent breadth, clinical investigations have traditionally prioritized anti-inflammatory and antioxidant rationales. To quantify this emphasis under contemporary reporting standards, we analyzed 117 trials first posted on or after 18 January 2017, the date on which the Final Rule for Clinical Trial Registration and Results Information Submission (42 CFR Part 11) took effect in the United States, standardizing requirements to improve data quality and comparability [136]. Among these studies, 50.4% (59/117) incorporated inflammatory and/or redox mechanistic coverage anywhere in the protocol (primary or secondary outcomes, rationale, biomarkers), with a component breakdown of 15.4% addressing both axes, 33.3% inflammation only, and 1.7% redox only.

Nonetheless, curcumin influences biological processes beyond conventional pathways, including glucose homeostasis, lipid metabolism, neuroplasticity, mitochondrial function, and gut barrier integrity, which are key elements of multifactorial chronic diseases. Emerging clinical findings suggest potentially meaningful benefits for glycemic control, hepatic steatosis, musculoskeletal function, and mood regulation, underscoring the opportunity to evaluate mechanistic domains not exclusively framed by immune or oxidative biology [7,17,59].

Taken together, these observations indicate that curcumin’s pharmacodynamic scope has been explored unevenly, potentially constraining systematic evaluation and perpetuating missed translational prospects. This trend likely reflects the convergence of cultural, scientific, and practical determinants that have favored mechanistic domains in which curcumin’s effects are most readily and reproducibly detected, particularly inflammation and oxidative stress. Contributing factors include: (i) centuries of use in Ayurveda, Traditional Chinese, and Unani medicine, which historically framed curcumin as an anti-inflammatory remedy [137]; (ii) well-recognized pharmacokinetic limitations, such as poor aqueous solubility, rapid conjugation, and extensive first-pass metabolism, that restrict systemic exposure [46,138] and, in turn, bias measurable effects toward proximal tissues or highly sensitive biomarker endpoints; and (iii) the strong dependence of clinical outcomes on formulation [135] and patient-specific variables, including microbiome composition [56,139], metabolic genotype [140], and concomitant medications [141].

### 4.2. Definitions and Scope

In this chapter, to better address curcumin’s pleiotropic nature, we focus on translational domains where inflammation or oxidative stress is not the primary therapeutic target. Although curcumin’s biology often intersects with redox and inflammatory pathways, we treat these as secondary mechanisms and selected primary outcomes accordingly. We therefore emphasize measures such as glycemic control, cognitive performance, metabolic function, hepatosteatosis, and microbiome-related outcomes.

We acknowledge, however, that defining *non-inflammatory* or *non-oxidative* domains is conceptually difficult, as metabolic, neurocognitive, and oncologic disorders frequently involve these processes to some degree, even when related biomarkers are absent or assessed only secondarily. A further interpretive challenge arises from the breadth of regulatory networks influenced by curcumin. Pathways such as AMPK activation [142], PPARγ modulation [143], mitochondrial signaling [144], and gut-microbiome interactions [145] align clearly with metabolic and neurocognitive outcomes and introduce little ambiguity.

In contrast, regulators such as Nrf2 and NF-κB occupy a conceptual overlap, as they are classically linked to oxidative and inflammatory responses yet also contribute to broader cellular homeostasis [146]. For this reason, we consider Nrf2 and NF-κB pleiotropic modulators or ancillary mechanistic contributors rather than strict determinants for trial inclusion.

### 4.3. Bioavailability, Formulation, and Pharmacokinetic Constraints

Oral curcumin exhibits very low systemic exposure. In patients with cancer, for example, a high dose of 3.6 g/day produced detectable concentrations within colorectal tissue, whereas only negligible levels were observed in the liver and other extraintestinal sites [119]. This limited systemic distribution was further confirmed in a dose-escalation trial, where free (unconjugated) curcumin remained undetectable in plasma at doses ≤ 8 g, and only minimal amounts, restricted to the nanomolar range, appeared at 10–12 g [50]. Consistently, a small crossover study using a standard 95% curcumin powder reported a peak-free curcumin concentration (C_max_) in plasma of approximately 0.3 ng/mL following a 400 mg oral dose containing 323 mg curcumin. However, when curcumin (64.6 mg) was administered to the same subjects in a liquid droplet micromicellar formulation, the normalized plasma exposure (AUC per milligram administered) increased by more than 500-fold relative to the standard powder, underscoring the potential of advanced delivery technologies to substantially enhance systemic diffusion [147].

To overcome curcumin’s bioavailability constraints, micelles, nanoparticles, phospholipid/micellar complexes, liposomes, and oil-based preparations have been developed to enhance solubility, absorption, and stability [135]. However, even with modern technologies, pharmacokinetic outcomes remain highly heterogeneous. For instance, an independent reassessment reported that the NovaSol^®^ formulation, which encapsulates curcumin in a lipid-based micellar carrier, can transiently elevate plasma curcumin concentrations to 6.7–38 nM, but these levels decline rapidly due to extensive phase II metabolism via glucuronidation and sulfation, underscoring persistent systemic limitations [118].

Collectively, these findings suggest that circulating concentrations often fall below those required to engage key molecular targets identified in vitro, potentially resulting in modest, variable, or inconsistent clinical outcomes. Thus, curcumin’s low and variable bioavailability represents a major translational bottleneck, warranting careful consideration of formulation strategies and pharmacokinetic parameters in clinical trial design and interpretation.

### 4.4. Multidimensional Biomarker Landscape and Clinical Implications

Biomarkers are increasingly employed in drug discovery to reduce translational risk, particularly in the context of multifactorial diseases or when developing agents with diverse and complex mechanisms of action [148]. For pleiotropic compounds such as curcumin, biomarker panels provide a critical framework for interrogating the underlying biology across multiple scales. This approach is especially relevant because curcumin’s broad mechanistic footprint necessitates multidimensional assessment and enables the identification of biomarkers intrinsically linked to its context-dependent biological effects.

Accordingly, biomarkers are widely incorporated into clinical studies that evaluate curcumin, most commonly in settings related to inflammation and oxidative stress. Trials frequently report changes in circulating cytokines (e.g., TNF-α, IL-6), C-reactive protein (CRP), and lipid peroxidation markers such as malondialdehyde (MDA), alongside modulation of antioxidant enzyme activities including glutathione peroxidase (GPx) and superoxide dismutase (SOD) [149,150,151]. Meta-analyses in populations with metabolic syndrome further indicate modest but statistically significant reductions in CRP, together with changes in physiological and metabolic parameters, such as fasting glucose, waist circumference, and HDL cholesterol, that are often interpreted within inflammatory or redox frameworks following curcumin supplementation [6,152]. Additionally, inhibition of COX2 and LOX enzymes, suppression of NF-κB, and downregulation of inflammatory cytokines such as TNF-α, IL-1, IL-6, and IL-8 mediate curcumin’s protective effects by reducing the severity of adverse effects (e.g., oral mucositis) associated with chemo- and radiotherapy [153].

While informative, conventional biomarkers are highly sensitive to baseline status, formulation, and analytical context, limiting their utility as standalone indicators of translational efficacy.

When primary clinical endpoints extend beyond inflammation and redox biology, reported biomarker changes encompass a much broader physiological spectrum, including metabolic, neurocognitive, microbiome-related, organ-level, musculoskeletal, and oncology-associated domains. This diversity aligns with curcumin’s pleiotropic pharmacology. Observed effects include improvements in lipid profiles, such as reductions in LDL-C and triglycerides accompanied by modest increases in HDL-C, as well as favorable changes in blood pressure and glycemic indices, including fasting glucose, HbA1c, and HOMA-IR [154,155].

In the neurocognitive domain, mechanistic biomarkers have been explored using advanced imaging and molecular readouts. In individuals without dementia, amyloid plaque and tau deposition assessed via FDDNP-PET imaging were associated with enhanced memory and attention following administration of Theracurmin^®^, a formulation optimized for intestinal absorption [156]. Similarly, in patients with moderate Alzheimer’s, treatment with CurQfen^®^ was linked to better cognitive and locomotor performance, accompanied by increased serum brain-derived neurotrophic factor (BDNF) and reductions in amyloid-β42 and tau protein [157], whereas in patients with schizophrenia, curcumin administered in capsules elevated BDNF levels but did not correspond to measurable gains in symptoms or cognitive functioning [158].

Beyond endogenous host biomarkers, the gut microbiota has emerged as a central mechanistic biomarker in human curcumin studies. Supported by extensive preclinical evidence, this perspective reflects the growing recognition that curcumin’s biological effects may be mediated, at least in part, through bidirectional interactions with the gut microbiome. In this context, curcumin can modulate microbial composition and functional activity, whereas microbial metabolism, in turn, influences curcumin’s bioavailability and bioactivity [56]. For example, in a double-blind, randomized, placebo-controlled trial in healthy adults, curcumin supplementation produced a significant increase in microbial diversity based on 16S rRNA gene sequencing, although responses varied markedly across participants. This variability, potentially influenced by the limited sample size and uncontrolled dietary factors, highlights the need for future investigations with larger cohorts and standardized diets to reduce interindividual and temporal fluctuations in microbiota profiles [159]. Notably, several clinical studies have incorporated microbiota-related endpoints to examine the effects of curcumin on gut-host interactions. These include analyses of short-chain fatty acid (SCFA)-producing taxa that increased in obese individuals undergoing weight-loss interventions, where curcumin was shown to enhance the relative abundance of *Bacteroidetes* and butyrate-producing taxa such as *Faecalibacterium prausnitzii* while simultaneously attenuating declines in probiotic *Actinobacteria* [160].

Moreover, in a 24-week randomized, double-blind, placebo-controlled trial in patients with non-alcoholic simple fatty liver disease, curcumin supplementation significantly reduced hepatic fat content and lowered the *Firmicutes*:*Bacteroidetes* ratio. This shift reflected both a decrease in the relative abundance of *Firmicutes* and an increase in *Bacteroidetes*, within which the study specifically reported an elevation of *Bacteroides*. Targeted metabolomics further revealed alterations in bile-acid metabolism, including higher serum deoxycholic acid and activation of bile-acid-sensing TGR5 signaling, pointing to a functional link between microbiota modulation and improved metabolic outcomes [161]. Additionally, microbiota profiling was incorporated into pediatric inflammatory bowel disease [162] and older adults with prediabetes, in which metagenomic sequencing was used to examine associations between microbial composition and clinical outcomes [163]. In this context, other microbiome-linked biomarkers capable of strengthening curcumin trials include indices of gut-barrier integrity (e.g., circulating or excreted zonulin) [164,165] and microbial metabolite signatures (e.g., fecal SCFAs) [166,167], which together provide complementary insight into colonic fermentation activity, microbial community function and composition, and host metabolic status.

In clinical trials evaluating organ function, curcumin is typically monitored through standard biochemical markers, such as liver enzymes, renal indices, lipid profiles, and glycemic parameters, which serve primarily as safety indicators and secondarily as efficacy measures. Across studies, these markers generally remain stable or show modest improvements, particularly in participants with pre-existing dysfunction or when enhanced formulations are used [6,155]. Notably, in critically ill patients with sepsis, a 10-day course of nano-curcumin improved inflammatory, oxidative, and endothelial biomarkers, while liver enzymes and creatinine showed minimal changes, consistent with a favorable safety profile [168].

In musculoskeletal conditions, clinical relevance is primarily defined by functional and patient-reported outcomes rather than biomarker normalization alone. Accordingly, endpoints such as pain scores, physical function, and health-related quality of life frequently exhibit short- to mid-term benefits following curcumin supplementation. Meta-analyses of randomized trials indicate that curcumin reduces creatine kinase and TNF-α, attenuates exercise-induced muscle damage, and improves range of motion and maximal voluntary contraction [169,170]. In knee osteoarthritis, systematic reviews report significant reductions in CRP and TNF-α with no significant differences in IL-6, IL-1β, ESR, and PGE_2_, aligning with observed improvements in pain and physical function [171,172].

In oncology-focused human studies, curcumin has been commonly evaluated in early-phase clinical trials aimed at assessing safety, pharmacokinetics, bioavailability, and feasibility rather than demonstrating anticancer efficacy. Although systemic concentrations of free curcumin remain low, conjugated metabolites are consistently detectable, supporting biological activity and justifying the use of biomarkers as secondary or mechanistic endpoints. For example, meta-analytic evidence across multiple cancer types indicates significant reductions in NF-κB and VEGF, with additional decreases in CRP, particularly when curcumin is administered alongside standard therapies. Biomarkers reflecting tumor-associated signaling pathways, including NF-κB, COX-2, EGFR, and various cell-cycle regulators, have also been assessed as proof-of-mechanism indicators rather than direct measures of therapeutic efficacy. Notably, nanotechnology-enhanced curcumin formulations demonstrate improved bioavailability and favorable safety profiles, with preliminary signals of symptom- or treatment-related toxicity modulation; however, in general, definitive conclusions regarding anticancer efficacy await larger, adequately powered trials [173,174,175].

Table 2 provides an overview of biomarkers and related clinical outcomes across the biological domains affected by curcumin.

**Table 2:** Biomarker Domains, Representative Measures, and Reported Clinical Effects Associated with Curcumin Supplementation.

Domain	Representative Biomarkers	Effects Reported	Primary Clinical Contexts	Notes	Refs.
Inflammation	CRP, TNF-α, IL-1β, IL-6, IL-8, ESR, PGE_2_, NF-κB	↓ CRP; ↓ TNF-α; ↓ IL-1,6,8	MetS, OA, cancer treatment tolerance	often involved to varying extents in other processes (e.g., metabolic, neuro-cognitive, and oncologic)	[6,149,150,151,152,153]
Oxidative Stress/Redox	MDA, GPx, SOD, COX-2, LOX	↓ MDA; ↑ antioxidant enzymes; ↓ COX-2; ↓ LOX	muscle damage, MetS, cancer treatment tolerance	influenced by redox state and assay conditions (also extended to other domains)	
Metabolic/Cardiometabolic	LDL-C, HDL-C, TG, fasting glucose, HbA1c, HOMA-IR, blood pressure, waist circumference	Improved lipid and glycemic markers	T2DM, NAFLD, metabolic syndrome	often interpreted with inflammation or redox shifts	[154,155]
Neurocognitive/Neuropathology	BDNF, Aβ42, tau, FDDNP-PET	↑ BDNF; ↓amyloid/tau; improved cognition	cognitive decline, Alzheimer’s disease, schizophrenia	formulation-driven (Theracurmin^®^, CurQfen^®^)	[156,157,158]
Microbiome	Microbial diversity (16S/metagenomics), SCFA-producing taxa, fecal SCFAs, bile acids (e.g., deoxycholic acid), *Firmicutes:Bacteroidetes* ratio, Bacteroides, *Faecalibacterium prausnitzii*, Actinobacteria, zonulin; TGR5 pathway	↑ microbial diversity; ↑ SCFA taxa; ↑ bile acid metabolism; ↓ F/B ratio; attenuates Actinobacteria decline; ↑ intestinal barrier integrity	healthy adults, NAFLD, obesity, weight-loss, IBD (pediatric), prediabetes (older adults)	high interindividual variability; microbial metabolism shapes effects	[56,159,160,161,162,163,164,165,166,167]
Organ Function	ALT, AST, creatinine, lipid and glycemic panels, endothelial markers	Neutral to favorable trends	sepsis, liver disease, supplementation trials	in sepsis trial: liver enzymes/creatinine largely unchanged; safety-oriented monitoring	[6,155,168]
Musculoskeletal	Creatine kinase (CK), CRP, TNF-α, IL-6, IL-1β, ESR, PGE_2_	↓ CK; ↓ TNF-α; ↓ exercise-induced muscle damage; ↑ ROM/MVC	exercise recovery, knee osteoarthritis	IL-6/IL-1β/ESR/PGE_2_ effects more variable; align with reduced pain and improved function	[169,170,171,172]
Oncology	NF-κB, COX-2, EGFR, VEGF, cell-cycle markers; plasma/tissue curcumin conjugates	↓ pathway activation; ↓ VEGF; ↓ CRP (when combined with therapies)	early-phase cancer trials	proof-of-mechanism endpoints; nanotechnology formulations improve bioavailability/safety	[173,174,175]

Summary of domains assessed in human clinical studies of curcumin, including representative molecular, biochemical, metabolic, neurocognitive, microbiome-related, and safety-oriented indicators. Reported effects reflect trends observed across randomized trials, meta-analyses, and formulation-specific investigations of curcumin or enhanced-bioavailability curcumin preparations. Biomarker responses vary according to baseline physiological status, curcumin formulation, dose, duration, and analytical methods. Arrows ↑ and ↓ denote increased or decreased values of the measured parameter. References correspond to studies cited in the text. Abbreviations: CRP, C reactive protein; TNF α, Tumor necrosis factor alpha; IL 1β/IL 6/IL 8, Interleukin 1 beta/6/8; ESR, Erythrocyte sedimentation rate; PGE_2_, Prostaglandin E2; NF κB, Nuclear factor kappa light chain enhancer of activated B cells; MDA, Malondialdehyde; GPx, Glutathione peroxidase; SOD, Superoxide dismutase; COX 2, Cyclo oxygenase 2; LOX, Lipoxygenase; LDL C, Low-density lipoprotein cholesterol; HDL C, High-density lipoprotein cholesterol; TG, Triglycerides; HbA1c, Glycated hemoglobin; HOMA IR, Homeostatic model assessment of insulin resistance; BP, Blood pressure; BDNF, Brain-derived neurotrophic factor; Aβ42, Amyloid beta 42; FDDNP PET, 2 (1 {6 [(2 [18F]fluoroethyl)(methyl)amino] 2 naphthyl} ethylidene) malononitrile positron emission tomography; SCFA, Short chain fatty acid; F:B ratio, *Firmicutes*:*Bacteroidetes* ratio; TGR5, Takeda G protein coupled receptor 5 (bile acid receptor); ALT, Alanine aminotransferase; AST, Aspartate aminotransferase; CK, Creatine kinase; ROM, Range of motion; MVC, Maximal voluntary contraction; EGFR, Epidermal growth factor receptor; VEGF, Vascular endothelial growth factor.

## 5. Concluding Remarks: The Tug-of-War Between Pleiotropy and Translation

Curcumin is widely recognized as a pan-assay interference compound (PAINS), a designation reflecting its propensity to generate misleading experimental signals through nonspecific reactivity, aggregation, redox cycling, and other complex physicochemical behaviors. These liabilities are especially problematic in high-throughput screening, where PAINS compounds are typically excluded to minimize false positives unless exceptional assay design, stringent interference controls, and rigorous interpretation are applied. Consequently, curcumin occupies a paradoxical position because it produces broad and often reproducible biological effects while simultaneously challenging conventional standards of pharmacological validation.

This paradox extends into human clinical research. Although curcumin modulates inflammatory, redox, metabolic, and signaling pathways, its translational success depends on the alignment of mechanistic plausibility, pharmacokinetic feasibility, and clinically meaningful endpoints. Emerging evidence suggests that curcumin’s therapeutic potential is best suited to conditions characterized by multifactorial dysregulation, such as metabolic syndrome, mild cognitive impairment, early neurodegenerative states, or hepatometabolic dysfunction, rather than diseases driven by a single dominant molecular target.

At the same time, curcumin’s pleiotropy introduces substantial variability into clinical outcomes. Effects differ according to formulation and bioavailability, administered dose and achievable tissue exposure, interindividual variation in microbiome composition and metabolic genotype, and heterogeneity in underlying disease biology. These factors contribute to the inconsistent magnitude, reproducibility, and durability of benefits reported across clinical studies [6].

Table 3 illustrates this pattern: several trials report beneficial outcomes, yet effect sizes vary widely and between-study heterogeneity remains substantial. Three recurrent challenges emerge. Specifically, curcumin tends to produce modest, distributed responses across multiple biological domains rather than strong effects within a single pathway; statistically significant results in individual trials often fail to replicate, with meta-analyses frequently noting substantial heterogeneity and limited robustness; and methodological inconsistencies, including heterogeneous formulations, insufficient statistical power, and suboptimal study design, further obscure interpretation and contribute to divergent conclusions.

Collectively, these factors define a persistent tension between curcumin’s mechanistic appeal and the practical constraints of experimental and clinical translation. This tension highlights the need to rethink how pleiotropic, context-dependent agents are evaluated across preclinical and clinical paradigms. Rather than prompting the dismissal of such chemistry, curcumin research appears well positioned to benefit from a *High-Input*, *Rational Integration* framework. This approach emphasizes information-dense, multimodal datasets (including orthogonal biochemical and cell-based assays), exposure-response PK/PD, metabolite profiling, and biologically congruent stress models, coupled with mechanistically grounded and context-aware interpretation [176].

Five operational pillars follow from this perspective. First, signal orthogonalization requires replication of key findings across assay platforms with independent failure modes, for example, biochemical versus phenotypic readouts or direct target engagement versus downstream pathway responses, paired with explicit interference controls. Second, exposure-anchored interpretation links observed effects to physiologically achievable concentrations of curcumin and its metabolites (free and conjugated), supported by standardized PK measurements, microdose-to-therapeutic crosswalks, and matrix-matched bioanalytics. Third, context calibration incorporates biological variability arising from microbiome composition, transporter expression, baseline inflammatory and redox tone, and disease stage to reduce overgeneralization of biologically contingent responses. Fourth, integrated biomarker strategies synthesize signals across metabolic, inflammatory, microbiome-related, and functional domains rather than over-focusing on single pathways. Finally, multivariate synthesis prioritizes network modeling, multivariate statistics, and machine-learning approaches over the isolated interpretation of individual endpoints (Table 4). Notably, this platform is increasingly compatible with modern computational approaches, including multimodal AI systems capable of integrating heterogeneous biochemical, cellular, pharmacokinetic, and systems-level datasets [177].

A complementary illustration of the *High-Input*, *Rational Integration* paradigm comes from a hypothetical program examining curcumin’s influence on hepatic energy-sensing pathways. Initial observations, such as modest activation of AMPK and shifts in mitochondrial substrate preference, are validated through orthogonal biochemical kinase assays, oxygen-consumption measurements, and cell-based reporter systems with appropriate interference controls. These signals are interpreted in the context of physiologically achievable concentrations of free and conjugated curcumin, supported by standardized PK and metabolite-back-conversion studies to avoid over-reliance on supra-physiological exposures. Experimental conditions are calibrated across biologically meaningful contexts, including variation in nutrient availability, hepatocyte metabolic state, and transporter expression, revealing that curcumin-responsive phenotypes emerge selectively under high-fat or high-substrate-load conditions. An integrated biomarker panel spanning lipid flux, mitochondrial efficiency, and intermediary metabolism replaces single-node readouts. Finally, multivariate analysis synthesizes these heterogeneous data layers, showing that the apparent AMPK-linked effects arise from a broader modulation of cellular energetic set points rather than direct, isolated target engagement.

Ultimately, curcumin’s translational trajectory signals a necessary shift in nutraceutical and drug-discovery paradigms, away from expectations of universal efficacy and toward a nuanced understanding of the contexts in which biological activity meaningfully translates to clinical benefit. With rigorous, mechanistically informed, and context-contingent evaluation, curcumin stands not as a warning or cautionary tale but a template for the rational development of therapeutic or semi-therapeutic agents.

**Table 3:** Human studies across multiple domains showcase the tug-of-war between curcumin’s pleiotropy and translational constraints.

Domain	Study Type & Population	Formulation Dose & Treatment Duration	Clinical Outcome(s)	Clinical Relevance & Evidence Quality	Refs.
Cognition	RCT; adults aged 51–84 (n = 40)	Theracurmin^®^ 180 mg/day 18 months	↓ amyloid, ↓ tau ↑ memory (SRT and BVMT-R) ↑ attention (Trail Making test part A)	the data prompted a larger study (n ~ 240), scheduled to start in 2026 [178] statistical significance observed for memory (WG *p* ≤ 0.01, BG *p* = 0.05) and attention (WG *p* < 0.0001, BG *p* = 0.04)	[156]
Cognition	meta-analysis of 9 RCTs (n = 501)	various (~0.8 g/day optimal) ≥24 weeks	↑ global cognitive function based on different assessment methods and parameters (MMSE, MCCB, ADAS-Cog, MoCA, CAQ, NIH toolbox)	improvement sustained ≥24 weeks; larger effect in Asian cohorts; efficacy increased with enhanced bioavailability substantial heterogeneity: I^2^ = 88.7%, *p* < 0.001	[179]
Anthropometrics	meta-analysis of 60 RCTs (n > 3650)	various 50–3000 mg/day (nano-curcumin to turmeric powder) 4 to 36 weeks	↓ BW, ↓ BMI, ↓ WC, ↓ BFP, ↓ leptin ↑ adiponectin	weight and obesity management, metabolic health, cardiovascular risk reduction high heterogeneity (I^2^ statistics across outcomes ranged from 78.5% to 96.3%)	[180]
Lipids	meta-analysis of 64 RCTs (n = 4051)	various 80–4000 mg/day (nano-curcumin to turmeric powder) 4 to 24 weeks	↓ TC, ↓ TG, ↓ LDL-c, ↑ HDL-c no effect on APO-A and Apo-B	lipid changes favorable for cardiovascular health very high heterogeneity for the lipid profile (I^2^ statistics across outcomes > 95%)	[181]
Hepatic	RCT; NAFLD patients (27 active vs. 28 placebo)	nano-curcumin 80 mg/day 16 weeks	↓ ALT, ↓ AST, ↓ LDH, ↓ GGT; no statistically significant differences in liver fibrosis or steatosis	improvement of certain aspects of liver function; further research is suggested to confirm long-term benefits and optimize dosing strategies statistical significance across liver enzymes: all *p*-values < 0.0001 (WG) and in the 0.001–0.043 range (BG)	[182]
Glycemic	meta-analysis of 28 RCTs (n = 2362)	whole CL (1000–2400 mg/day) CL extract (300–1950 mg/day) bioavailability-enhanced (80–1000 mg/day) 4–16 weeks	↓ fasting blood glucose (FBG) ↓ HbA1c	positive impact on glycemic control substantial heterogeneity: I^2^ (FBG) = 75.8%, I^2^ (HbA1c) = 83%,	[183]
Microbiome	RCT; healthy adults (n = 30; 3 arms)	Curcumin C3 Complex^®^ or turmeric tablets (1000 mg) plus black pepper extract Bioperine^®^ (1.25 mg) 8 weeks	curcumin group: ↑ 69% turmeric group: ↑ 7% placebo group: ↓ 15% (assessed by 16S rDNA sequencing using Shannon entropy indices)	increased microbial diversity, beneficial for metabolic, immune, and inflammatory functions) alpha diversity not statistically significant (*p* = 0.8), suggesting inconsistent or unreliable within-group differences	[159]

Human studies and meta-analyses across different domains were selected to illustrate the contrast between beneficial clinical outcomes linked to curcumin administration and substantial heterogeneity, as reflected in the *p*-values and I^2^ statistics reported in the Clinical relevance & evidence quality column. Arrows ↑ and ↓ denote increased or decreased values of the measured parameter. Abbreviations and technical terms: Study design & population: RCT, Randomized Controlled Trial; n, Sample size; NAFLD, Non-Alcoholic Fatty Liver Disease. Formulation, Dose, & duration of treatment: Theracurmin^®^, A highly bioavailable colloidal curcumin formulation; Curcumin C3 Complex^®^, A standardized curcuminoid extract containing curcumin, demethoxycurcumin, and bisdemethoxycurcumin; Bioperine^®^, A black pepper extract standardized to piperine, used to enhance bioavailability; CL, Curcuma longa (turmeric). Clinical outcomes: SRT, Selective Reminding Test; BVMT-R, Brief Visuospatial Memory Test-Revised; Trail Making Test part A, A neuropsychological test assessing processing speed and attention; MMSE, Mini-Mental State Examination; MCCB, MATRICS Consensus Cognitive Battery; ADAS-Cog, Alzheimer’s Disease Assessment Scale-Cognitive Subscale; MoCA, Montreal Cognitive Assessment; CAQ, Cognitive Assessment Questionnaire; NIH Toolbox, Standardized cognitive assessment battery developed by the U.S. National Institutes of Health; BW, Body Weight; BMI, Body Mass Index; WC, Waist Circumference; BFP, Body Fat Percentage; TC, Total Cholesterol; TG, Triglycerides; LDL-c, Low-Density Lipoprotein Cholesterol; HDL-c, High-Density Lipoprotein Cholesterol; Apo, Apolipoprotein (A or B); FBG, Fasting Blood Glucose; HbA1c, Glycated Hemoglobin A1c; ALT, Alanine Aminotransferase; AST, Aspartate Aminotransferase; LDH, Lactate Dehydrogenase; GGT, Gamma-Glutamyl Transferase; 16S rDNA sequencing, Sequencing of 16S ribosomal DNA to identify bacterial taxa; Shannon entropy index, A metric of microbial alpha-diversity; Clinical relevance & evidence quality: WG, Within-Group comparison; BG, Between-Group comparison; *p*, *p*-value (statistical significance); I^2^, I-squared statistic (degree of heterogeneity in meta-analysis).

**Table 4:** Summary of the Five Operational Pillars within the *High-Input*, *Rational Integration* Framework.

Pillar	Core Concept	Operational Focus	Primary Objective
Signal orthogonalization	Validate findings across assays with independent failure modes to minimize artifacts	Use biochemical and phenotypic assays; combine direct target-engagement assays with downstream pathway readouts; include explicit PAINS/interference controls	Reduce false positives and ensure observed activity is not due to assay artifacts
Exposure-anchored interpretation	Link biological activity to physiologically or clinically achievable concentrations of curcumin and its metabolites	Standardized PK; profiling of free and conjugated metabolites; microdose-to-therapeutic crosswalks; matrix-matched bioanalytics	Ensure mechanistic plausibility and avoid claims based on non-physiological concentrations
Context calibration	Account for biological variability to avoid overgeneralizing context-dependent responses	Integrate microbiome composition, transporter expression, baseline inflammatory/redox tone, and disease stage	Identify responder subgroups and reduce variance arising from biological heterogeneity
Integrated biomarker strategies	Use multi-domain biomarker panels instead of single-pathway markers	Combine metabolic, inflammatory, redox, microbiome-related, and functional biomarkers	Capture pleiotropic biological responses and avoid misleading single-marker interpretations
Multivariate synthesis	Apply integrative analytical methods to interpret distributed biological activity	Network modeling; multivariate statistics (e.g., Principal Component Analysis, Partial Least Squares); machine learning approaches	Provide coherent interpretation of pleiotropic effects without overweighting isolated endpoints

## Figures and Tables

**Table 1:** Examples of marketed drugs affected by curcumin through CYP3A modulation, efflux transporter inhibition, or combined mechanisms.

Drug	Primary Mechanism(s)	Key Observed Outcome(s)	Experimental Model	Key Refs.	Confirmatory and Conflicting Evidence
Midazolam	↓ intestinal P-gp and CYP3A ↑ hepatic P-gp and CYP3A ↔ renal P-gp and ↑ renal CYP3A	↑ Cₘₐₓ & ↑ AUC, (decreased oral clearance)	Rats (60 mg/kg curcumin/4-day gavage)	[110]	inconsistent with CYP3A activation shown in another rat model [111]; no PK/PD changes observed in a human study [125]
Everolimus	↑ CYP3A metabolism > ↓ P-gp	↓ Bioavailability	Rats (50 & 100 mg/kg)	[111]	confirmed in two human case studies [126], reinforcing clinical and therapeutic significance.
Sulfasalazine	↓ BCRP	↑ Absorption and systemic exposure	Preclinical (mice) and Human clinical data	[116,117,127]	confirmed in preclinical and clinical settings; administering curcumin 4–5 days before sulfasalazine appears to eliminate the interaction, likely due to curcumin’s low bioavailability and rapid clearance. This suggests a potential strategy for managing drug interactions in patients consuming curcumin as a supplement or dietary component, with important safety implications.
Celiprolol	↓ intestinal P-gp	↑ Cₘₐₓ & ↑ AUC, (increased absorption)	Rats (60 mg/kg curcumin/4-day gavage)	[110]	curcumin’s effect on pharmacokinetics was observed with sustained dosing rather than acute intake; current evidence is limited to preclinical models and remains unconfirmed in humans.
Paclitaxel	↓ intestinal P-gp and CYP3A	↑ Cmax & AUC (1.4–1.5×); ↓ clearance and distribution; ↑ oral bioavailability and cytotoxicity	Swiss Mice (100 mg/kg curcumin for 4 days, orally)	[128]	altered PK in rabbits and tissue distribution changes in rats were also demonstrated [129]; human trials did not assess PK but showed improved efficacy (objective response rate 53% with curcumin vs. 33% without) and a favorable safety profile, likely due to controlled dosing and route of administration (intravenous) [130].
Fexofenadine	cocrystals of Fexofenadine HCl (FEX) with Curcumin and Piperine as coformers	↑ permeability (2.68-fold) and ↑ dissolution rate	ex vivo goat intestinal tissue	[131]	fexofenadine, a P-gp substrate, shows improved bioavailability when co-crystallized with nutraceuticals.
Atorvastatin	↓ CYP3A2	↑ plasma exposure	male Sprague-Dawley rats (hyperlipidemia model) (30 mg/kg curcumin via gavage, PK tests performed after 7-day treatment)	[132]	current evidence shows no added lipid-lowering benefit from combining curcumin with atorvastatin, while posing potential herb-drug interaction risks. No human PK or efficacy studies are available, highlighting safety concerns without a demonstrated therapeutic advantage.
Tacrolimus	modulation of CYP3A enzymes [110]	↑ plasma exposure	single human case study	[133]	direct link between turmeric intake and altered tacrolimus metabolism, with acute kidney injury resolving after turmeric withdrawal → the need to reconcile turmeric/curcumin use in tacrolimus-treated patients. Similar mechanistic findings were also observed in preclinical studies [134].

Drugs were selected based on (i) published in vivo or human pharmacokinetic evidence; (ii) mechanistic clarity regarding CYP3A4 and/or ABC transporter involvement; (iii) clinical or translational significance based on therapeutic context. Drug classification/application: Midazolam, Sedative/Anxiolytic—Used in anesthesia, procedural sedation, and treatment of seizures; Everolimus, Oncology/Immunosuppressant—Commonly used for certain cancers (breast, kidney, neuroendocrine tumors) and to prevent organ transplant rejection; Sulfasalazine, Anti-inflammatory/Rheumatology & Gastroenterology—Used for inflammatory bowel disease (IBD) and rheumatoid arthritis; Celiprolol, Cardiovascular (Beta-blocker)—Indicated for hypertension and sometimes for vascular disorders; Paclitaxel, Oncology (Chemotherapy)—Used for breast, ovarian, lung cancers, and others; Fexofenadine, Antihistamine (Allergy treatment)—For allergic rhinitis and chronic urticaria; Atorvastatin, HMG-CoA reductase inhibitor (Statin)—Lipid-lowering agent used to reduce total cholesterol, LDL-C, and triglycerides indicated for hyperlipidemia and prevention of cardiovascular disease; Tacrolimus, Calcineurin inhibitor (Immunosuppressant)—Prevention of organ transplant rejection (kidney, liver, heart) and used in some autoimmune conditions. It works by inhibiting T-cell activation. Arrows ↑, ↓, and ↔ denote increased, decreased, or unchanged values of the measured parameter. Abbreviations: P-gp, P-glycoprotein (ABCB1); BCRP, breast cancer resistance protein (ABCG2); CYP nomenclature follows that used in the original studies. Human isoform names (e.g., CYP3A4) are retained when drugs are used as translational probe substrates, even in rodent models, whereas species-specific isoforms (e.g., CYP3A2) are reported when explicitly defined as such by the authors.

## Data Availability

No new data were created or analyzed in this study. Data sharing is not applicable to this article.
